# Mini-documentaries and the invited gaze: an effective way of cross-cultural communication

**DOI:** 10.1007/s40636-023-00262-y

**Published:** 2023-03-09

**Authors:** Yang Zhuofan, Luo Jun, Lin Na

**Affiliations:** 1 Academy for International Communication of Chinese Culture, Beijing Normal University, Beijing, China; 2 School of Foreign Languages, Southwest Jiaotong University, Chengdu, China

**Keywords:** Invited gaze, Subjectivity, Site-specific, Cognitive schema

## Abstract

The nature of mini-documentary is to be true to the story. But being true is not necessarily the replicate of the original, instead, the director can restructure the story based on the original and make it more artistic and enjoyable for the audience. It would be ideal if viewers from different walks of life can take pleasure in interpreting the film in their own ways. A recap of the documentaries about China both from home and abroad reveals either the bias or superficialness to a large extent. The long-term and systematic representation of the people and stories about China is what we need to shed the bias and the misrepresentations. This paper takes the Looking China Youth Film Project as the case to analyze a cross-cultural communication model that enables the international young filmmakers to experience and know about China.

The nature of mini-documentary is to be true to the story. But being true is not necessarily the replicate of the original, instead, the director restructures the story based on the original and makes it more artistic and enjoyable for the audience. It would be ideal if viewers from different walks of life can take pleasure in watching and perceiving the film. Both the director and the sponsor can decide the angles they present the story to the audience. A recap of the documentaries about China both from home and abroad reveals either the bias or superficialness when telling China stories, such as the politically hyped narratives of the western politics toward China, the pursuit of social trend by some domestic media in the name of spreading Chinese culture, and the creation for sensations and commercial gains by vested interest groups. The long-term and systematic representation of the people and stories about China is what we need to shed the bias and the misinterpretation. This paper takes the *Looking China* Youth Film Project as the case to analyze a cross-cultural communication model that enables the international young filmmakers to experience and know about China.

*Looking China* is a cross-border Chinese culture experiencing program that is sponsored by the Beijing Normal University. It kicked off in 2011 and has been held for 12 consecutive years. Every year, about 100 international young filmmakers are invited to experience the Chinese culture and shoot documentaries based on their own observations and points of view. Chinese student volunteers are selected as producers to co-create 10-min-long documentaries with the young filmmakers under a general theme set for each year. The impact of COVID-19 after its outbreak in 2020 has posed difficulties for the implementation of *Looking China*, but the organizers have worked out new ways to continue the program by either inviting international students living and studying in China as the filmmakers, or using virtual meeting platforms for filmmakers overseas to co-create documentaries with their Chinese producers. *Looking China* is special in the way it gives full play to international young filmmakers’ potential and self-initiative so that they can shoot films with distinct personal styles and through their gaze toward China and the world. What’s more, the documentaries get better media exposure internationally after the filmmakers voluntarily share their works on social media or other platforms. By far, the works of *Looking China* have gained widespread recognition at film festivals and on TV channels abroad. They were broadcast 4002 times by i CiTi TV, a TV station with 35 million viewers in the US, and received over 10 million hits on the Internet. Some of the works were screened at film festivals such as the Bilbao International Festival of Documentary and Short Films, the International Student Film Festival of Pernambuco, the BFI London Short Film Festival, the Kolkata International Film Festival and the Tel Aviv International Student Film Festival (TISFF), and won awards of various kinds. There were also screening tours on campus of some overseas universities. The influence of the project is broadened from bottom-up with effective communication models.

This paper probes into the significance of cross-cultural communication through local culture experience programs with the focus on three key elements related to the creation of documentaries, subjectivity, site-specific and cognitive schema. First, the non-profit and non-governmental nature of *Looking China* enables the filmmakers to pursue the topics they take real interest in. Second, most of the filmmakers and the producers who are students at universities find it easier for them to build up rapport with each other due to similar age and educational level, in turn, smooth cooperation yields great works. Third, the filmmakers and producers manage to approach their interviewees with ease as their identity avails to the establishment of empathy and communicability with the subjects. They get the chance to stay close with the family and social circles of the protagonists. They are able to film people’s lives by having equal dialogues that empower the ordinary people to open up their hearts in front of the camera. The interviewees in many cases are people whose voices are seldom heard under a lofty theme or a grand social background and whose lives are much ignored by the mainstream. By *Looking China*, their stories are unfolded by images of familiar daily routines which become extraordinary through the narratives of international young filmmakers. The gap of communication and culture exchange is bridged in the way that the films are no longer sponsorship-centered but people-oriented.

The striking difference of the young filmmakers from documentary makers of other types lies in their motives and perspectives. The *Looking China* filmmakers are invited to know China and portray China with their own eyes while some filmmakers of other types are intruding into a culture. Cecilia Mello in her paper *Transit, ethnography and the camera gaze: a cross-cultural perspective* proposed the concept of “the invited gaze”, saying “The young filmmakers who will look at China, opening their cameras to the country’s reality, are there under an invitation. They are, therefore, not intruders, but guests. This undermines any trace of the predominantly ethnographic gaze that is guided by an investigative and sometimes imposed force.” Arun Gupta, a scholar from India and who was also an international supervisor of *Looking China*, described his understanding of “the invited gaze”, mentioning that the international young filmmakers are not only cameramen, but participators of a cross-cultural experience project. They shoot films with a new cognitive schema with a participatory role, instead of spying on or imposing force on a culture from a distance.

## Subjectivity of international young filmmakers

On the invitation of *Looking China*, the international young filmmakers land on the Chinese culture and deliver their creations based on their own cultural background and usually breaking the stereotypes about China. In contrast to Chinese young filmmakers, mainstream media outlets and professional individual filmmakers, their films showcase unique perspectives, deep insights and creative ways of shooting. Their films are imbued with personal styles and mirror their understanding of Chinese culture and people. This paper first analyzes how the subjectivity of filmmakers is turned into persuasive powers that woo the audience.

Rich researches have been devoted to the study of subjectivity of film making. Peng Xiaojun, a Chinese scholar, believes that the subjectivity of a documentary attests to the motive and creativity of the filmmaker with a variety of elements throughout the film. The filmmakers of *Looking China* are taken as the Other, and they deliver unique and intuitive narratives by bringing in their own gazes. From the eye of the Other, they avoid one-sidedness and arrogance in cross-cultural communication, opening up a new path to tell stories by cinematographic language. The *Looking China* filmmakers observe the daily life of the protagonists, describe it with fresh angles and marks of the self-gaze, and present the films about China with a renewed gaze of international youth.

### Gaze of the Other: revisit the forgotten daily routines

Following a daily routine that repeats itself day by day, we may lose interest in the people and surroundings that are so familiar. The numbness of sight, hearing, smell and touch looms in, especially when we perceive daily life and public spaces such as subways, streets and parks as dull existence. The vitality of international young filmmakers broke the dullness and helped us rediscover the life and inner heart that we always turn a blind eye to. By the camera lens, the familiar things in daily life were endorsed with new meanings and took on a new look. The aesthetic and cultural connotations of daily life were re-conditioned and brought in front of camera gaze. Jean Paul Sartr, philosopher of existentialism, saw the gaze as the battleground for the self to define and redefine itself; we become aware of our self-egos as subject only when confronted with the gaze of the Other and become aware of our self-egos as object. Since the second half the 20th century, in the circles of western literary criticism and cultural criticism, to gaze implies more than to look at–it signifies a psychological relationship of power, in which the gazer is superior to the object of the gaze. Zizek proposed that to practice the gaze is to enter a personal relationship with the person being looked at. The Žižekian analysis of cinema focuses on the production of enjoyment in spectatorship. The subjective position of the spectator is developed prior to cinematic spectatorship and thus films do not interpellate individuals as subjects. The Gaze in this paper is foremost a recognition that the Other perspective is absent from documentaries about China, so the focal point is to facilitate the connection between the Other and the Self. The Gaze is the key to bringing the people, the customs and the culture that we often take for granted back into spotlight and in front of the camera by introducing the role of the Other. Documentaries of *Looking China*, such as *Beijing, The Painter’s Quest, Jiao Tong Teahouse, Maple at Night, Connection, Everlasting Longing*, tell stories behind ordinary daily scenes such as Hutongs, tea houses, bridges, public transportation, etc. from the eye of the Other. For example, what is the real intention of an Israeli youth in Xi’an exploring the beauty of ancient Chinese poems from the Tang Dynasty? The director narrated his encounter with a Chinese family, his feelings on campus, at the night street and in the hustle and bustle of city life. The poetry was given new meanings in the relationship of mother and son, between lovers, and between friends, and the inquisitive gaze of the film was unveiled in front of the audience: the poems of the Tang Dynasty are rooted in the interpersonal relationship, in the inner world of the people and in the cultural legacy of Chinese from generation to generation. In the film *The Painter’s Quest*, the director Emanuel Hänsenberger from Switzerland grew up learning music and painting from his father and was keen on the topic of Hutong after he began his journey in Beijing. With the help of the Chinese student-volunteer, he made a film based on dialogues with three painters who are representative of different age groups, life experiences and sets of values, revealing their memories, observations and visions of Hutongs. Beijing as an international metropolitan was quite different from the widely perceived images such as CBDs, the Tian’anmen Square, the Great Wall, the Forbidden City and the Summer Palace. With the gaze of an international young filmmaker, the city showed multiple facets revealed by the beauty of Hutongs, as well as the love and care of the people, and he reflected on the superficial renovation of a city over its originality.

### Interpretation of the Other: thick description

Geertz borrowed the phrase “thick description” from the philosopher Ryle and drew a comparison with thin description. He explained a thick description is a description of human social action that describes not just physical behaviors, but their context as interpreted by the actors as well, so that it can be better understood by an outsider. A thick description typically adds a record of subjective explanations and meanings provided by the people engaged in the behaviors. As in the case of *Looking China*, the international young filmmakers are not giving thin description of the subjects, but an interpretation with their fresh gaze. For example, in the film *Painting a Forest,* the Israeli filmmaker Shauly Melamed traveled to Saihanba of Hebei Province, and used a large number of old photos and video materials to picture the stereotype of the western world toward China. He invited forest guards of two generations to sit together and have a dialogue of mutual understanding, showing the efforts of desertification control from generation to generation. The film didn’t praise the work of forest guards directly, but unfurled the daily life of those people and the relationship between humans and the desert, which is a very intuitive gaze from the filmmaker himself.

Typically, the international young filmmakers from a low-context culture tend to get every detail related to the film under control and work hard on digging into the topic and the subject within a limited time in order to fully interpret and retell the story from the high-context Chinese culture to the world with their inquisitive gaze. They develop the overlapping gaze of self-awareness and close observation of China which bypasses the culture gap between the west and the east and enables the international audience to know about China.

### Generation Z: unique perspectives

The Generation Z refers to the people who were born in the late 1990s in a world dominated by the Internet. They show distinctive features in using the media language, expressing the narratives and forming new ways for receiving information. They possess the unique identity while generating a special power in cross-cultural communication. On various new media platforms, the Generation Z of China perceived by western media is different from the elder generations and has a potential influence on the future of the world.

The author of the paper found that the young filmmakers of *Looking China* have their own and common interest in the topics of China.

They firstly focused on families of diverse ethnic groups in China. Children, father, mother, grandfather and grandmother constitute a relatively close intergenerational relationship in Chinese family culture and also reflect the family inheritance in China's multi-ethnic regional culture. The ethnic culture inheritance based on the family unit is shown in three aspects in their documentaries: first, they filmed the families’ inheritance based on some traditional customs, usually the elders ("old people") of the family acted as the inheritors of the customs, and the young ("children") learned from the elders; Second, they filmed family inheritance based on crafts; The third is that they filmed family based inheritance education, such as the family education of minority ethnic languages.

They secondly foucused on the destiny of mankind in their films when they were in China. At present, China is in the process of urbanization development. These international young filmmakers' sharpened "tentacles" soon reached out to problems related to the development of urbanization, especially topics about the fate of mankind.

## Site-specific experience and film cooperation

This paper investigates how the documentaries become site-specific as the second focal point. “Documentary makers ‘intrude into’ the life of others by being on the site, similar to the fieldwork of anthropologists. They then enter into a paradox of being both an outsider and an insider” (Zhuoting 2015). There are similar concepts proposed about being site-specific, for example, according to the article “Reflection on Space: Site-Specific Art” published in the Shanghai Art Review based on a project of Beijing Film Academy, “Site-specific means to be on the spot with specific time, location, people and events, including both the geographical and natural dimensions and the historical and humanistic dimensions.” The purpose of carrying out fieldwork is to collect evidence by being there and from the perspective of I witness to satisfy the pursuit of authenticity based on top-down experience (Zhuoting 2015). The site-specificness of this paper contains two meanings. First, it means the information receiver can gain hands-on experience by being there on the site during cross-cultural communication. Second, it is a process of building mutual understanding between the information receiver and the local people. Context and subjectivity play a key role for information receivers in constructing a cognitive paradigm of different cultural perspectives by on-site experiences with the local people and in local culture. For example, the main character of the documentary *Mement*o is Sami who is a Palestinian international student in Central China Normal University. He was the first Arabian who recovered from COVID-19 in the world. By recalling his own experience of being infected and cured during the epidemic, Sami told the story of what he felt during the epidemic. His interpretation of the fight against the epidemic set a context with which he demonstrated what he saw of China. The film *Country Wagons* was created via online cooperation by Jorge Eduardo Tort Oviedo from Mexico and the Chinese producer-volunteer. The filmmaker adopted narratives of animation and diagrams and combined them with post-editing of footage shot on site to show the transformation of families in China’s urbanization process, as well as the life of left-alone elderly people in the countryside and the connotation of “filial piety” in the modern time. The creation of the film was deeply influenced by the filmmaker’s previous 17-day on-site experience in the countryside of Guangxi, China back in 2017. Being on site makes it possible for international filmmakers to see in order to believe, and tell stories about China with their true feelings and real experience.

Site-specific experience is first and foremost based on the geographical location, especially the urban and rural areas with historical and cultural heritages. People’s daily life can be contextualized to trigger a deep understanding toward the local history, environment and lifestyle. A new context is built with the merger of meanings and details through the invited gaze. Site-specific experience is direct and first-hand with the filmmaker’s personal reflections based on his or her observation, comprehension and interaction with the subject on site.

The site-specific witness is authentic and first-hand. The documentary *Portraits for You* shot in 2016 in Xinjiang is about a little girl and her relation with the culture of her nationality, the Xibo nationality. The shooting of the little girl’s life is site-specific, and with the images of daily scenes, the film exerts an influence on the audience and their cinematographic experience. The documentary *The Last Small Village* produced in the 2017 *Looking China* Guangxi reveals the beauty of Zhuang ethnic minority’s life. The local culture of the minority people is showcased through the ecology, the traditional way of farming, the family life and the folk songs. The discovery of a real China by the international filmmaker is realized through his site-specific experience which is translated into interaction with local people. The empathy of the audience is aroused within this site-specific context and the film becomes more vivid and touching.

Site-specific experience is also to feel and grasp the essence of things. The subject of the documentary *Jiao Tong Teahouse*, a production of 2019 *Looking China* Chongqing, builds upon its theme of local life in Chongqing by the filmmaker’s observations which are expressed by narratives of a diary. The sweetness and sorrow of daily life, as well as the key role of tea houses in the local culture, are captured by the filmmaker who creates a site-specific context for the audience. *Watch People*, a documentary from the 2017 *Looking China* Fujian, is about the repair of watches from every detail, revealing a heart-warming story of the protagonist’s self-healing in face of life’s difficulties. Director Christian Grobbelaar from South Africa said, “When you are shooting a documentary, it’s like an opportunity to grow in emotions. Your vision is broadened because you can get involved into the life of those total strangers. You get to know them and their feelings. This is an adventurous journey as you see beautiful and new things which you would have never imagined in the past.” He is telling the true feeling of lots of the international filmmakers involved in *Looking China*. The documentary *Agen* about the religion of the Tibetans is a creation during the 2019 *Looking China* Qinghai. The site-specific experience is the best way to capture the uniqueness of the local people and culture, and arouse the audience’s empathy with the Tibetans’ religion and emotions. The *Three Flavours of Chongqing* produced during 2018 *Looking China* Chongqing presents the very local features characterized by the flavors of nature, history and lifestyle. The filmmaker’s experience on site is the key to decoding the actual link between spicy food and the climate. The site-specific filming connects the plots naturally and promotes the camera effect for the viewers.

## International young filmmakers and their subjects: co-building a cognitive schema

Taylor and Crocker (1981) put forward the concept of schema as a cognitive structure that consists in part of a representation of some defined stimulus domain. The schema contains general knowledge about that domain, including specification of the relationships among its attributes, as well as specific examples or instances of the stimulus domain (Taylor and Crocker 1981). The international young filmmakers are conceiving their cognitive schema by close contact with their subjects based on observation and immersion into the local life.

In line with Taylor’s definition of schema, there are three layers of cognition pattern of international young filmmakers, namely, their knowledge about China such as knowing chopsticks, kongfu, Taichi, Confucianism, bike sharing and Alipay, their understanding of the connection of Chinese elements such as the relationship between Taichi and Confucian thoughts, and their unique personal experience in China such as Italian youth Andrea’s interpreting of hotpot culture. The schema is co-built with the subjects in the films, referring to either protagonists or life scenes under a specific background.

Based on the etic and emic perspective in anthropology, the *Looking China* filmmakers make observations either as an insider or an outsider, both contributing to the construction of a new schema.

### An outsider perspective

This refers to the films made when the international young filmmakers are not directly involved in the subjects’ daily life and only observe them from a distance. This perspective used to be taken as the biased interpretation of the story hyped with superficial pursuit of camera effect.

But director Juss Saska from Estoni in the documentary *Bird’s Nest* adopted an outsider angle to produce the same stunning sensation. He recalled, “The first time I set my foot in the Longchang Apartment, I was shocked because it felt like another world among the high-rises of Shanghai. The feeling was so real and vivid that I was anxious to record everything there from daybreak to late night. I feel that I don’t need any words to explain what it is like and what is out there. I just need to observe quietly and transfer this feeling to the audience. Silent images can be more powerful than words.” His experience sheds light on the construction of the cognitive schema by both filmmakers and their subjects. The filmmakers deliberately keep a distance from the subjects without interfering with their life while capturing everything via camera lens, from which they present the films with sharpened understanding of the lifestyle, social status and values of the subjects.

### An insider perspective

With an invited gaze, the international young filmmakers get a close look at the subjects’ daily life, and are able to record their interactions, dialogues and interviews. The insider’s view narrows the gap between directors and their protagonists, and is conducive to the expression of true emotions.

Gao Feng, a documentary filmmaker and Executive Dean of AICCC, quoted a poem *Joyful to See My Cousin then Said Goodbye to Him* by Li Yi, a poet from the Tang Dynasty. It tells the surprise reunion of the poet and his cousin after many years of departure. They could not recognize each other at first, but soon picked up childhood memories and had warm conversation until late at night. Once people get to know each other, they find something in common and get closer to each other. The international filmmakers are close enough to the subjects’ life and are not intruders any more. They develop the mutual understanding with the protagonists and both sides show sincerity to enable the smooth communication, which in turn, helps build the cognitive schema of the filmmakers.

### A passionate gaze

The international filmmakers can both maintain a distance from the subjects and get involved in their daily life based on the needs, and this ensures them both objectivity and empathy while shooting on site. Albert Maysles, an American documentary filmmaker in the Direct Cinema style, regarded this type of shooting as embedded with “the gaze” (Lewis 2000).

Filmmaker Francesco Eramo from Italy in his documentary *The Keeper of the Bell* kept a distance from the protagonist on purpose, believing this is the better way to shoot the real life of the bell keeper. He said “I did lots of research on the bell keeper and was happy to get to his home for talks and interviews. But soon I realized a distance was necessary when shooting my film because I wanted to make it different from others. What I needed was to keep an observing eye.”

In *Looking China* project, the international young filmmakers shoot their documentaries on the site after entering into the life, culture and circles of the protagonists through either an insider’s or an outsider’s perspective. In both ways, the role of the subjects is undeniable for the formation of a cognitive schema.

To sum up, *Looking China* has contributed a lot to the exploration of a feasible path for better cross-cultural communication. The analysis in this paper has been built on expounding the subjectivity, the site-specific nature and the construction of a cognitive schema with the aim to promote the understanding of cross-cultural communication and media management. This is still a discussion that has unresolved issues and further study needs to be continued such as in the aspects of different historical, cultural and social background. But researches of this nature are of significance to understand cross-cultural communication of China in a wider perspective.
